# The Antecedents of Positive Emotion and Continuous Usage of In-Flight Meals with Respect to Food Quality Using Structural Equation Modeling

**DOI:** 10.3390/foods13162622

**Published:** 2024-08-21

**Authors:** Won Seok Lee, Joonho Moon

**Affiliations:** 1 Department of Tourism and Recreation, Kyonggi University, Suwon 443760, Republic of Korea; lws79877@gmail.com; 2 Department of Tourism Administration, Kangwon National University, Chuncheon 24341, Republic of Korea

**Keywords:** in-flight meal, menu diversity, familiarity, temperature, nutrition, presentation

## Abstract

This work aimed to explore the food quality attributes of in-flight meals and to examine the antecedents of positive emotion and continuous usage of these meals. As a subdimension, this study uses multiple attributes: menu diversity, familiarity, temperature, nutrition, and presentation. Another purpose of this work is to examine the moderating effect of menu diversity on the relationship between nutrition and continuance usage. A survey via clickworker was used to collect the data for this work. There were 317 valid observations for statistical inference. This study used a structural equation model to test the hypotheses, and the Hayes process model macro 1 was adopted to test the moderating effect. The results showed that all independent variables other than familiarity significantly accounted for positive emotion. Moreover, all of these attributes had a positive impact on continuous usage. This work unveiled a significant moderating effect of menu diversity on the relationship between nutrition and continuance usage. This research elucidates the literature by clarifying the influential attributes of emotion and continuous usage intention in the domain of in-flight meal products and discussing practical implications.

## 1. Introduction

Previous research has shown that in-flight meals are critical for evaluating overall service in airline businesses [1,2]. Additionally, scholars have reported that in-flight meals are consumed in unique contexts, such as high altitudes and uncomfortable conditions [3,4]. It indicates that the consumer’s perspective of the in-flight meal is likely to vary in a unique manner. However, scholars have rarely the investigated consumer’s perception of the in-flight meal. Considering these findings, investigating the characteristics of in-flight meals is valuable. Such outcomes might play an important role in improving the airline service experience.

This work first scrutinizes the elements of food quality in the case of in-flight meals. According to Peri [5], the definition of food quality varies depending on the context. Because in-flight meals are consumed in unusual conditions, it is worth exploring the critical attributes for food quality. This review of the literature revealed five attributes: menu diversity [6,7], familiarity [8,9], temperature [10,11], nutrition [12,13], and presentation [14,15]. Next, this work aims to examine the impact of in-flight meal food quality on positive emotion and continuous usage. In previous works, positive emotion [16,17] and continuous usage [18,19] have been adopted as the explained attributes, so this work adopts both elements to achieve the research goal.

This study aims to examine the moderating effect of menu diversity on the relationship between nutrition and continuance usage. Scholars have documented that the nutritional value of food has become a more important element for consumer decision-making because individuals have become more interested in health conditions through the enhancement of living conditions [12,13,20]. It suggests that airlines are likely to offer the nutritional option to consumers, which might result in choice overload problems. Previous research has documented that choice overload is related to negative consumer evaluation [21,22], and people often consider nutritional food unappetizing [23,24]. This could be applied to the case of in-flight meals. Such a problem might offset the returns an airline receives for better service. Moreover, airlines might need to increase the options by adding nutritional options in the case of in-flight meals, it might be necessary to examine whether adding a nutritional menu is effective or not. To scrutinize such an argument, this work is to inspect the moderating effect of menu diversity on the influence of nutrition on continuance usage. This research aims to answer the following questions:

Research question 1: How food quality of in-flight mean is defined?
Research question 2: How does food quality affect consumers’ positive emotions and their intention to continue using in-flight meal services?

Overall, the aims of this work are as follows: (1) explore the definition of food quality in the area of in-flight meals using multiple attributes and (2) determine the effects of food quality on consumer behavior by employing positive emotion and continuous usage as dependent variables. Another aim of this work is to explore the moderating effect of menu diversity on the relationship between nutrition and continuance usage. Given that the definition of food quality can vary significantly depending on the context [5], it is important to establish a clear definition specific to the domain of in-flight meals. Furthermore, it is crucial to assess whether the identified attributes adequately explain consumer behavior. Hence, this research contributes to the literature by documenting the definition of in-flight meals and presenting the effect of food quality on passenger behavior. Additionally, this work sheds light on the literature by identifying the relationship between nutrition, continuance usage, and menu diversity. Furthermore, this work is valuable because the results can be used as a reference for improving the quality of in-flight meals.

## 2. Review of the Literature and Hypotheses Development

### 2.1. Continuance Usage

Previous studies have demonstrated that continuous usage—characterized by the ongoing relationship between consumers and suppliers—is a form of loyalty behavior [25,26]. Prior studies have also shown that continuous usage is related to businesses having a greater market share and sales growth [18,19]. Many scholars have adopted continuous usage as a main attribute. For instance, Ramos [27] chose continuous usage as the main attribute for exploring the users of food delivery apps. Chong et al. [28] also explored the determinants of continuous usage in the case of electronic commerce. Inan et al. [29] scrutinized mobile banking users using continuous usage as the dependent variable. In addition, Abdul-Halim et al. [25] examined e-wallet users’ behavior by employing continuous usage as the outcome variable. The literature review shows that continuous usage has been extensively studied by numerous researchers.

### 2.2. Positive Emotion

Emotion is defined as a mood caused by external stimuli, and researchers have claimed that emotion is a kind of instinctive consumer reaction [30,31,32]. Essén and Wikström [33] reported that emotion is a reaction to the quality of goods and services. Many previous studies have examined emotion because it is regarded as an immediate reaction by consumers [16,17]. Wu and Shen [34] explored the antecedents of positive emotion in the area of food service businesses. Li et al. [35] also examined the determinants of positive emotion in the context of peer-to-peer accommodation services. Altinay et al. [30] investigated the determinants of positive emotion in patients. Additionally, Im et al. [36] investigated elements that influence positive emotion in solo diners. Thus, this literature review indicated that numerous researchers have studied the characteristics of positive emotion as the explained variable.

### 2.3. Food Quality in the Area of In-Flight Meals

Food quality is defined in various ways because it is difficult to define using a single word [5,37]. This study attempts to define food quality in the context of in-flight meals by extending the existing theoretical frameworks. Through an extensive literature review, the research aims to identify and delineate the subdimensions of food quality in in-flight meals from the perspective of consumers. First, this research suggests menu diversity. Offering diverse menu options allows consumers to choose food based on their preferences [38,39,40]. Additionally, scholars contend that offering diverse options is beneficial from the consumer’s perspective because the choice is more likely to meet their flavor requirements [6,7,41]. The next area of this work is familiarity. Familiarity is defined as the extent to which individuals are accustomed to goods and services [42,43,44]. Because food choice depends on individual experience and cultural diversity, offering familiar food allows consumers to feel more comfortable [8,9]. Moreover, the extant literature indicates that individuals prefer familiar goods because consumers dislike uncertainty in consumption, which could be applied to the case of in-flight meals [45,46,47]. Xu and Zeng [48] found that food familiarity significantly affects tourists’ decision-making. The third element of this work is temperature. Temperature is a sensory factor in food quality because taste evaluation varies with temperature [10,11]. Indeed, researchers have stated that food temperature is a worthwhile point for examining consumer behavior because it is related to food safety [10,11]. Liu and Lee [49] and Jaja and Iroegbu [10] suggested that food temperature is imperative for consumer behavior in the food service domain. By synthesizing the existing literature, it can be inferred that food temperature is crucial both as a sensory element and for ensuring safe consumption. The fourth domain of this research is nutrition. Cranage et al. [50] reported that nutrition is a cue for assessing food quality from the perspective of consumers. The market has become increasingly interested in healthy food because food consumption is directly linked with a healthy life [12,13]. Specifically, scholars contend that food quality is determined by nutritional value [12,51]. Indeed, the extant literature indicates that healthy food is a critical research area because of its popularity [20,52]. The last area of this study is presentation. Presentation refers to the aesthetically attractive element of food [53,54]. Food presentation is related to the first impression of food, which makes consumers pay attention to the food [14,15]. Previous studies have suggested that food presentation is a critical factor in the food product industry, serving as an effective marketing tool [15,55,56]. Based on the literature review, the subdimensions of food quality in the context of in-flight meals include menu diversity, familiarity, temperature, nutrition, and presentation. Overall, this work presents five attributes as subdimensions of food quality in the case of in-flight meals focusing on the prior studies’ argument that food quality is likely to be defined in varied manners depending on the context [5,37]. This research utilizes five attributes as subdimensions to define the food quality of in-flight meals and investigates how these dimensions influence consumers’ perceptions.

### 2.4. Hypotheses Development

The first piece of this work is the association between food quality and emotion. In this work, the subdimensions of food quality are defined as menu diversity, familiarity, temperature, nutrition, and presentation. Walsh et al. [57] and Cardello and Jaeger [58] argued that food quality significantly impacts emotion, as good food enhances consumers’ overall well-being. Köster and Mojet [59] and Prinyawiwatkul [60] alleged that food plays a significant role in building a positive mood for consumers. Zhong and Moon [61] also revealed the positive effect of food quality on positive emotions. Ouyang et al. [62] demonstrated that food quality is an influential attribute of positive emotion. It can be inferred that the subdimensions of food quality are likely to enhance consumers’ perceptions and intentions. This research thus proposes the research hypotheses as follows:

**Hypothesis** **1a:** *Menu diversity positively affects positive emotion.*

**Hypothesis** **2a:** *Familiarity positively affects positive emotion.*

**Hypothesis** **3a:** *Temperature positively affects positive emotion.*

**Hypothesis** **4a:** *Nutrition positively affects positive emotion.*

**Hypothesis** **5a:** *Presentation positively affects positive emotion.*

The next area of this work is the relationship between food quality and continuous usage, which is regarded as a loyalty attribute. Previous research has revealed that food quality positively impacts the intention to continue using a vendor [63,64]. Bihamta et al. [65] also found a positive influence of food quality on loyalty by exploring restaurant customers in hotels. Additionally, Zhong and Moon [61] demonstrated that food quality is a critical determinant of loyalty in the domain of fast-food businesses. They implied that continuance usage is likely to be positively influenced by food quality-related attributes: menu diversity, familiarity, temperature, nutrition, and presentation. Therefore, this study proposes the following research hypotheses:

**Hypothesis** **1b:** *Menu diversity positively affects continuous usage.*

**Hypothesis** **2b:** *Familiarity positively affects continuous usage.*

**Hypothesis** **3b:** *Temperature positively affects continuous usage.*

**Hypothesis** **4b:** *Nutrition positively affects continuous usage.*

**Hypothesis** **5b:** *Presentation positively affects continuous usage.*

### 2.5. Moderating Effect of Menu Diversity on the Impact of Nutrition on Continuance Usage

Previous research has argued that offering nutritional menu options is a crucial business strategy in the food service sector, as consumers place greater value on their health with economic development [13,52]. However, offering a healthy menu is likely to expand the range of options, which could, in turn, place a burden on consumers’ information processing during decision-making. Namely, a nutritional option is likely to be related to the choice overload problem. The definition of choice overload is that too many options cause adverse effects on consumer evaluation of the product or service [22,66]. Previous research stated that choice overload is common in the consumer behavior area, and the findings indicated that consumers are dissatisfied with their choice under excessive options [21,22,67]. Moreover, scholars addressed that the belief that healthy food lacks flavor is a common misconception, which can influence consumer behavior in food choices [23,24,68]. Integrating the review of the literature, it can be inferred that offering a diverse menu with more nutritional options is likely to cause undesirable consumer appraisal because too many options with healthy menus are linked with perceptions of the food being unflavored and negative perceptions such as dissatisfaction and regret. Regarding the review of the literature, this research proposes the following research hypothesis:

**Hypothesis** **6:** *Menu diversity significantly moderates the effect of nutrition on continuance usage.*

### 2.6. Research Model

**Figure 1:**
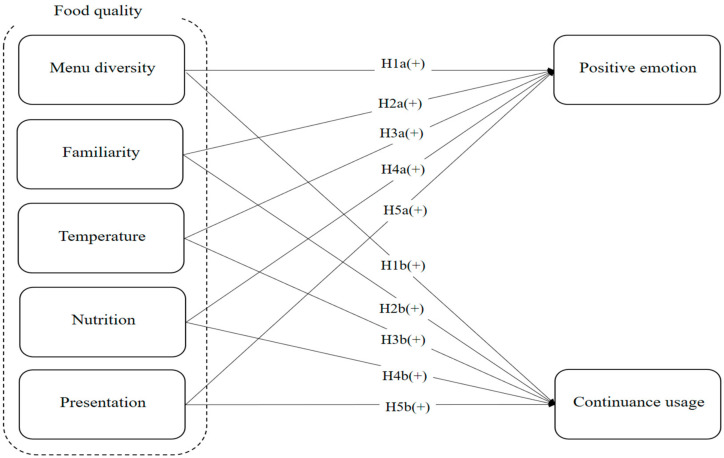
Research model.

Figure 1 illustrates the research model, which includes five explanatory variables: menu diversity, familiarity, temperature, nutrition, and presentation. Additionally, this work employs two explained attributes: positive emotion and continuous usage. All the independent variables have a positive effect on both the dependent variables.

**Figure 2:**
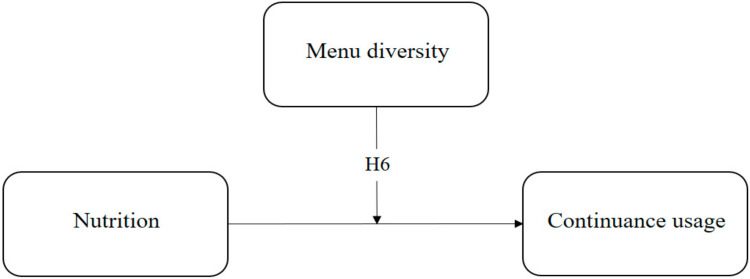
Research model for moderating effect.

Figure 2 shows the moderating effect of menu diversity. Nutrition is the independent variable, and continuance usage is the dependent variable. Moreover, menu diversity significantly moderates the relationship between nutrition and continuance usage.

## 3. Method

### 3.1. Description of Measurement Items

Table 1 shows the descriptions of the measurement items. For the measurement, this research employed a five-point Likert scale (1 = strongly disagree, 5 = strongly agree). Additionally, only familiarity was measured using three items, while the other constructs had four items. The measurement items for nutrition [12,13], presentation [15,56], positive emotion [30,36], and continuous usage [25,29] were derived from prior studies. In addition, for this research, two research experts in the hospitality and tourism discipline were consulted to measure menu diversity, familiarity, and temperature. In terms of definition, menu diversity refers to how individuals perceive various options for in-flight meals. Familiarity is defined as how individuals evaluate an in-flight menu based on their prior experience/knowledge of the menu options. Temperature is defined as the degree to which individuals sense the food temperature to be appropriate. Nutrition is defined as how individuals assess food for health condition improvement. Additionally, ‘presentation’ refers to the aesthetic attribute of food from the passengers’ perspective. Positive emotion is defined as a feeling toward in-flight meal consumption. Continuous usage is the customers’ intention to keep consuming in-flight meals in the future. Furthermore, the survey collected demographic information, including age, sex, employment status, monthly household income, and annual frequency of in-flight meals.

### 3.2. Data Collection and Instruments for Analysis

The clickworker panel service (https://www.clickworker.com/) was adopted for this research. Numerous studies have employed clickworker data collection tools [69,70], so it can be inferred that the data quality of clickworker is reliable for statistical inference. The data were collected between 1 February and 7 February 2024. This research employed simple random sampling, as the survey notice was made publicly accessible. Initially, 364 observations were collected. This research included a screening question to choose participants who had experience with in-flight meals. Moreover, this research focused on Americans due to the large number of airlines in the U.S. that offer in-flight meal services. Thus, 47 out of 364 individuals who had no experience with in-flight meals were eliminated from this work. Consequently, 317 valid observations were used for the data analysis in this study.

Table 2 shows the demographic information of the survey participants. Regarding gender, the numbers of males and females were 61 and 256, respectively. Table 2 shows the information on age (20s or younger: 80, 30s: 127, 40s: 83, 50s: 21, and older than 60: 6) and monthly household income (under USD 2500: 55, USD 2500–4999: 93, USD 5000–7999: 65, USD 7500–9999: 36, and over USD 10,000: 68). Table 2 also shows the annual frequency of in-flight meal use (less than 1 time: 38, 1–2 times: 184, 3–5 times: 69, and more than 5 times: 26).

In this work, frequency analysis was performed to examine the demographic information of the survey participants. Then, confirmatory factor analysis was conducted to test the convergent validity and reliability of the measurements. The extant literature has shown that convergent validity is ensured through the following thresholds: factor loading: 0.5, construct reliability (CR) value: 0.7, and average variance extracted (AVE): 0.5 [71,72]. The mean and standard deviation of the constructs in this work were computed. A correlation matrix was employed to assess the relationships between variables and to ensure discriminant validity. Previous studies have shown that discriminant validity is acceptable when the square root of the AVE is greater than the correlation coefficient [71,72,73]. In this study, a structural equation model for hypothesis testing was executed by applying the following rule for goodness of fit: Q (CMIN/degree of freedom) < 3, RMR (root-mean-square residual) < 0.05, RMSEA (root-mean-square error of approximation) < 0.05, GFI (goodness-of-fit index) > 0.8, NFI (normed fit index) > 0.8, RFI (relative fit index) > 0.8, IFI (incremental fit index) > 0.8, TLI (Tucker-Lewis index) > 0.8, and CFI (comparative fit index) > 0.8 [71,72,73]. Covariance-based maximum-likelihood-based structural equation modeling was adopted for the data analysis in this work. Furthermore, this research tested the moderating effect of menu diversity using the Hayes Process Macro model 1 with bootstrapping = 5000. Then, this research performed a median split analysis to scrutinize the moderating effect of menu diversity using four groups: (1) low nutrition × low menu diversity, (2) high nutrition × low menu diversity, (3) low nutrition × high menu diversity, and (4) high nutrition × high menu diversity

## 4. Results

### 4.1. Results for Convergent and Discriminant Validity

Table 3 shows the results of the confirmatory factor analysis. The AVEs and CRs were greater than the threshold. Moreover, all the factor loadings were greater than 0.5. Moreover, the goodness of fit was acceptable compared to the threshold values (χ^2^ = 681.208, df = 303, χ^2^/df = 2.248, RMR = 0.046, GFI = 0.854, NFI = 0.923, RFI = 0.911, IFI = 0.956, TLI = 0.949, CFI = 0.956, and RMSEA = 0.063). Overall, convergent validity was ensured. Additionally, Table 3 presents descriptive statistics for the following attributes: menu diversity (mean = 2.916, SD = 1.000), familiarity (mean = 3.262, SD = 1.081), temperature (mean = 3.761, SD = 0.933), nutrition (mean = 3.027, SD = 0.948), presentation (mean = 3.575, SD = 0.950), positive emotion (mean = 3.195, SD = 1.076), and continuous usage (mean = 3.258, SD = 0.974).

Table 4 is the correlation matrix. The discriminant validity of the constructs was ensured by comparing the diagonal values with the off-diagonal values. Continuous usage was positively correlated with menu diversity (r = 0.594, *p* < 0.01), familiarity (r = 0.394, *p* < 0.01), temperature (r = 0.467, *p* < 0.01), nutrition (r = 0.620, *p* < 0.01), presentation (r = 0.603, *p* < 0.01), and positive emotion (r = 0.765, *p* < 0.01). Additionally, positive emotion was positively correlated with menu diversity (r = 0.672, *p* < 0.01), familiarity (r = 0.361, *p* < 0.01), temperature (r = 0.507, *p* < 0.01), nutrition (r = 0.701, *p* < 0.01), and presentation (r = 0.693, *p* < 0.01).

### 4.2. Results of Hypothesis Testing

Table 5 shows the results of the structural equation model. Positive emotion was positively influenced by menu diversity (β = 0.594, *p* < 0.05), temperature (β = 0.467, *p* < 0.05), nutrition (β = 0.620, *p* < 0.05), and presentation (β = 0.603, *p* < 0.05). Additionally, continuous usage was positively affected by menu diversity (β = 0.594, *p* < 0.05), familiarity (β = 0.394, *p* < 0.05), temperature (β = 0.467, *p* < 0.05), nutrition (β = 0.620, *p* < 0.05), and presentation (β = 0.603, *p* < 0.05). Given the values of the goodness-of-fit indices (χ^2^ = 583.194, df = 336, χ^2^/df = 1.736, RMR = 0.025, GFI = 0.918, NFI = 0.937, RFI = 0.929, IFI = 0.972, TLI = 0.969, CFI = 0.972, and RMSEA = 0.040), the outcomes from the structural equation model were statistically significant.

Table 6 depicts the results of the moderating effect of menu diversity on the relationship between nutrition and continuance usage. The model was statistically significant regarding the F-value (*p* < 0.05). The results revealed a significant and negative effect of Nutrition × Menu diversity (β = −0.089, *p* < 0.05) on continuance usage. Therefore, H6 is supported.

**Figure 3:**
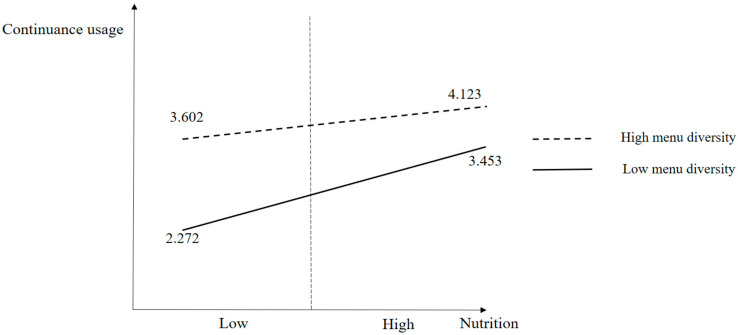
Moderating effect of menu diversity on the effect of nutrition on continuance usage.

Figure 3 shows the results of the median split analysis. The results present the mean value of four groups (mean_low nutrition×low menu diversity_ = 2.272, mean_high nutrition×low menu diversity_ = 3.453, mean_low nutrition×high menu diversity_ = 3.602, and mean_high nutrition×high menu diversity_ = 4.123). The low nutrition and low menu diversity group showed the lowest mean value, while the high nutrition and high menu diversity group showed the highest mean value.

## 5. Discussion

This work investigated the factors influencing positive emotion and continuous usage in the area of in-flight meals. Even though in-flight meals could become an important aspect of the airline service, scant studies have been explored. This research thus attempted to streamline such a research gap. By following the approach of the extant literature, this work employed both positive emotion and continuance usage as explained attributes [27,28,74]. Based on a literature review, this research proposed five elements to operationalize food quality in the area of in-flight meals; five attributes, namely, menu diversity, familiarity, temperature, nutrition, and presentation, were identified. Considering descriptive statistics, consumers assessed the temperature of in-flight meals most positively, while the appraisal of menu diversity was somewhat skeptical. The results showed that menu diversity, adequate temperature, nutrition, and presentation significantly affected both positive emotion and continuous usage. Moreover, the results revealed that familiarity only exerted a positive effect on positive emotion. Specifically, customers assigned more value to having more options for their in-flight meals. Additionally, it can be inferred that customers view familiar food as a crucial component of in-flight meals, as it reflects diverse cultures and experiences from their perspective.

Next, this research revealed that temperature is an essential attribute of in-flight meal consumption because this consumption is less comfortable than that of on-the-ground meals. Furthermore, the results revealed that the nutritional value, which is related to improved health conditions, is critical in the case of in-flight meals because passengers inside an aircraft might become more exhausted and require more energy during travel without concern for their health condition. Furthermore, the results revealed that the aesthetical piece is a crucial point for a better perception of in-flight meals because it might be related to the first impression of the food. All things considered, the results validated the findings of prior studies in that menu diversity [7,41], familiarity [8,9], temperature [10,11], nutrition [75,76], and presentation [14,15,77] are essential in the case of in-flight meal service from the viewpoint of consumers.

Considering the reasons for nonsignificance, familiarity might result in somewhat negative emotions such as boredom. In terms of magnitude, presentation appeared to be the most influential attribute of positive emotion. Moreover, familiarity appeared to be the strongest determinant of continuous usage of in-flight meals. Additionally, this research tested the moderating effect of menu diversity on the association between nutrition and continuance usage. The results showed the significant moderating impact of menu diversity. The findings of this study corroborate previous research, demonstrating that choice overload leads to adverse outcomes in consumer decision-making by increasing effort, stress, and confusion [21,22,67]. Specifically, the results suggest that menu diversity may be a double-edged sword for healthy menus, as it can lead to choice overload. Additionally, consumers might have misperceptions about the nutritional menu with more varied options because sometimes consumers often wrongly perceive healthy food as unappetizing.

## 6. Conclusions

This work contributes to the literature by documenting food quality in the area of in-flight meals. Building on Peri’s argument [5] that the definition of food quality is heterogeneous, this research explored how food quality is defined in the context of in-flight meals. Although in-flight meals are imperative for airline service, the extant literature has rarely explored the food quality of in-flight meals. To fill this research gap, this research suggested five subdimensions for food quality in the domain of in-flight meals. Moreover, this research contributes to the literature by revealing the significant connection between suggested attributes and explained variables: positive emotion and continuous usage. This might support the external validity of this work for determining the relationships among food quality, positive emotion, and continuous usage [61,63,64]. This work also contributes to the literature by identifying the moderating impact of menu diversity on the association between nutrition and continuance usage. As already addressed in the extant literature [67,68,78], this research shed light on the literature by further demonstrating the likelihood of choice overload and its negative aspects in the area of in-flight meals. Such an outcome might be worthy because it validates the notion of choice overload in the case of in-flight meals.

This work has managerial implications. Airlines might be able to consider developing more menus for consumers. Moreover, airline managers might invest more in enhancing food familiarity, which might be achieved by improving the food quality in one’s own country rather than by providing exotic food to customers. Through consumer analysis, airlines might be able to identify their main consumers’ characteristics in terms of food culture and background, and airlines might dedicate their constrained resources more efficiently. In addition, managers might need to focus on food containers for in-flight meals to maintain the optimal temperature for eating. Furthermore, airline managers might need to allocate more resources to provide customers with nutritional food, which might be achieved through labeling and nutrition disclosure efforts. Airline management might also need to contemplate how to present food aesthetically because it could enhance passengers’ perceptions. These efforts are likely to result in enhanced positive emotions and sustained usage. Because airline resources are limited, airline managers might be able to prioritize familiarity and presentation because these attributes appear to be more influential than other attributes on positive emotion and continuous usage, respectively. Furthermore, the nutritional option could be considered for in-flight meal service, if airlines offered a single menu to the consumers. However, airline managers may need to exercise caution when offering a diverse array of healthy menu options. While providing a variety of choices can be beneficial, it may also lead to choice overload, which can negatively impact consumer decision-making and increase operational costs for the airline. By doing so, airline managers might achieve a better position in their in-flight meal food quality.

This work has limitations. First, the target of this study was limited to U.S. customers. Researchers might be able to consider samples from various countries. Moreover, this study explored only the linear relationships between attributes, and future research might be able to test more complex relationships between variables.

## Figures and Tables

**Table 1:** Description of the measurement items.

Construct	Code	Item	Reference
Menu diversity	MD1	The airline offered diverse in-flight meal menus.	Consulting
	MD2	The airline prepared various in-flight meal menu options.	
	MD3	The airline allowed me to choose from many in-flight meal menu options.	
	MD4	The airline provided diverse in-flight meal menu options.	
Familiarity	FM1	I was familiar with the in-flight meal.	Consulting
	FM2	The in-flight meal was known to me.	
	FM3	I was used to the in-flight meal.	
Temperature	TM1	The temperature of in-flight meal was adequate.	Consulting
	TM2	The in-flight meal had a good temperature.	
	TM3	The in-flight meal was offered with an adequate temperature.	
	TM4	The temperature of the in-flight meal was appropriate.	
Nutrition	NT1	The in-flight meal was nutritional.	French et al. [12]; Losasso et al. [13]
	NT2	The in-flight meal’s nutrition promoted my health condition.	
	NT3	The nutritional value of in-flight meal was adequate.	
	NT4	The in-flight meal’s nutrition was excellent.	
Presentation	PT1	The presentation of the in-flight meal was organized.	Szocs and Lefebvre [15]; Lin and Wang [56]
	PT2	The presentation of the in-flight meal looked good.	
	PT3	The presentation of the in-flight meal was neat.	
	PT4	The presentation of the in-flight meal was nice.	
Positive emotion	PE1	I was happy with the in-flight meal.	Altinay et al. [30]; Im et al. [26]
	PE2	I was joyful about the in-flight meal.	
	PE3	I was pleased with the in-flight meal.	
	PE4	I was delighted with the in-flight meal.	
Continuance usage	CU1	I intend to use the in-flight meal again.	Abdul-Halim et al. [25]; Inan et al. [29]
	CU2	I am willing to pay for the in-flight meal again.	
	CU3	I have an intention to purchase the in-flight meal again.	
	CU4	I am going to choose the in-flight meal again.	

**Table 2:** Demographic information (*n* = 317).

Characteristics	Frequency	Percentage
Sex		
Male	61	19.2
Female	256	80.8
Age		
20–29 years old or younger	80	25.2
30–39 years old	127	40.1
40–49 years old	83	26.2
50–59 years old	21	6.6
Older than 60	6	1.9
Monthly household income		
Under USD 2500	55	17.4
USD 2500 and USD 4999	93	29.3
USD 5000 and USD 7499	65	20.5
USD 7500 andUSD 9999	36	11.4
Over USD 10,000	68	21.5
Annual using frequency of in-flight meals		
Less than 1 time	38	12.0
1~2 times	184	58.0
3~5 times	69	21.8
More than 5 times	26	8.2

**Table 3:** Confirmatory factor analysis results.

Construct (AVE)	Code	Loading	Mean (SD)	CR
Menu diversity (0.725)	MD1	0.863	2.916 (1.000)	0.913
	MD2	0.828		
	MD3	0.799		
	MD4	0.911		
Familiarity (0.649)	FM1	0.716	3.262 (1.081)	0.846
	FM2	0.846		
	FM3	0.847		
Temperature (0.856)	TM1	0.855	3.761 (0.933)	0.959
	TM2	0.975		
	TM3	0.986		
	TM4	0.877		
Nutrition (0.703)	NT1	0.832	3.027 (0.948)	0.904
	NT2	0.802		
	NT3	0.813		
	NT4	0.902		
Presentation (0.772)	PT1	0.799	3.575 (0.950)	0.931
	PT2	0.906		
	PT3	0.903		
	PT4	0.902		
Positive emotion (0.807)	PE1	0.885	3.195 (1.076)	0.943
	PE2	0.900		
	PE3	0.920		
	PE4	0.887		
Continuance usage (0.771)	CU1	0.766	3.258 (0.974)	0.930
	CU2	0.916		
	CU3	0.915		
	CU4	0.905		

Note: *p* < 0.05. Goodness-of-fit indices: χ^2^ = 681.208, df = 303, χ^2^/df = 2.248, RMR = 0.046, GFI = 0.854, NFI = 0.923, RFI = 0.911, IFI = 0.956, TLI = 0.949, CFI = 0.956, RMSEA = 0.063. CR denotes construct reliability, and AVE stands for average variance extracted.

**Table 4:** Correlation matrix.

	1	2	3	4	5	6	7
1. Menu diversity	0.851						
2. Familiarity	0.306 *	0.805					
3. Temperature	0.381 *	0.295 *	0.925				
4. Nutrition	0.612 *	0.393 *	0.434 *	0.838			
5. Presentation	0.530 *	0.307 *	0.505 *	0.645 *	0.878		
6. Positive emotion	0.672 *	0.361 *	0.507 *	0.701 *	0.693 *	0.898	
7. Continuance usage	0.594 *	0.394 *	0.467 *	0.620 *	0.603 *	0.765 *	0.878

Note: * *p* < 0.01.

**Table 5:** Results of hypothesis testing.

Path	β	t Value	*p* Value	Results
Menu diversity → Positive emotion	0.308	5.93 *	0.000	Supported
Familiarity → Positive emotion	0.046	1.10	0.269	Not supported
Temperature → Positive emotion	0.114	2.82 *	0.005	Supported
Nutrition → Positive emotion	0.257	3.97 *	0.000	Supported
Presentation → Positive emotion	0.319	5.53 *	0.000	Supported
Menu diversity → Continuance usage	0.131	2.59 *	0.010	Supported
Familiarity → Continuance usage	0.286	4.55 *	0.000	Supported
Temperature → Continuance usage	0.108	2.21 *	0.027	Supported
Nutrition → Continuance usage	0.205	2.64 *	0.008	Supported
Presentation → Continuance usage	0.232	3.39 *	0.000	Supported

Note: * *p* < 0.05. Goodness-of-fit indices: χ^2^ = 583.194, df = 336, χ^2^/df = 1.736, RMR = 0.025, GFI = 0.918, NFI = 0.937, RFI = 0.929, IFI = 0.972, TLI = 0.969, CFI = 0.972, and RMSEA = 0.040.

**Table 6:** Results of testing moderating effect of menu diversity.

Variables	β	t Value
Constant	−0.244	−0.56
Nutrition	0.760 *	5.26
Menu diversity	0.693 *	4.34
Nutrition × Menu diversity	−0.089 *	−1.94
F value	88.59 *	
R^2^	0.4592	
Conditional effect of focal predictor		
Menu diversity		
2.00	0.581 *	7.68
3.00	0.491 *	7.56
4.00	0.402 *	4.88
Test of unconditional interaction		
Δ R^2^	0.006	
F-value	3.96 *	

Note: Dependent variable: continuance usage * *p* < 0.05.

## Data Availability

The data presented in this study are available on request from the corresponding author due to privacy.
